# Human Skin Permeability Prediction by Machine Learning for Transdermal Drug Delivery

**DOI:** 10.3390/medicina62101929

**Published:** 2026-10-07

**Authors:** Seunghwan Oh, So Hyun Ahn

**Affiliations:** 1 Department of Materials Science and Engineering, College of Engineering, Yonsei University, 50 Yonsei-ro, Seodaemun-gu, Seoul 03722, Republic of Korea; osh020927@yonsei.ac.kr; 2 Ewha Medical Research Institute, School of Medicine, Ewha Womans University, Seoul 03760, Republic of Korea; 3 Ewha Medical Artificial Intelligence Center, School of Medicine, Ewha Womans University, Seoul 03760, Republic of Korea

**Keywords:** skin absorption, administration, cutaneous, analgesics, opioid, quantitative structure–activity relationship, machine learning

## Abstract

*Background and Objectives*: The dose a transdermal patch delivers is governed by the drug’s permeability coefficient (Kp) across skin, and the reference Potts–Guy equation assumes log Kp is linear in lipophilicity and molecular weight. Whether that form holds across the whole of the public human data, and how far such a model generalises to a new therapeutic class, is unknown. *Materials and Methods*: Two open human skin permeation databases were merged and standardised, yielding 732 permeability records for 327 compounds, 40 of them analgesics. Fifteen two-dimensional descriptors were computed. Linear, ridge, random forest and gradient boosting models were compared by cross-validation, a held-out test set and leave-analgesics-out extrapolation, with every fold and split defined on compounds. *Results:* Predicted log Kp rose steeply with lipophilicity but plateaued above an octanol–water partition coefficient of about 2.5, a saturation confirmed by accumulated local effects. The apparent decline above a molecular weight of about 220 g mol^−1^ spanned only 0.44 log units, lay within its bootstrap interval, and is reported as model-derived. The bilinear Potts–Guy equation could not represent this form (R^2^ = −0.172 pooled). A random forest reached R^2^ = 0.312 on the held-out set (median 0.383 across 200 resplits), outperforming Potts–Guy (*p* = 0.006). Epidermis-only analysis raised R^2^ from 0.213 to 0.443, a large effect that loses significance once fold dependence is corrected (*p* = 0.059). With the analgesic class withheld, a two-descriptor linear model beat both ensembles; all 40 lay inside the applicability domain, yet predictions were compressed where fentanyl, sufentanil and morphine sit. *Conclusions*: Lipophilicity shows a saturating relationship with skin permeability that a linear equation cannot represent. Achievable accuracy is limited at least as much by variability between independent studies of the same compound as by molecular description, and interpolation within the sampled chemical space does not imply generalisation to a new class.

## 1. Introduction

Chronic pain affects a substantial minority of adults and is among the leading causes of years lived with disability worldwide; in the United States 50.2 million adults, or 20.5 per cent, report pain on most or every day, and low back pain, the single largest contributor to years lived with disability, affected an estimated 619 million people globally in 2020 [1,2]. Systemic analgesia is effective but carries dose-dependent gastrointestinal, renal and central nervous system toxicity, and the oral route exposes the whole body to achieve a local or steady effect. Topical and transdermal analgesia offers an alternative in which the drug is presented at or through the skin, and Cochrane syntheses support the efficacy of several topical formulations in musculoskeletal and neuropathic pain [3,4].

Transdermal patches occupy a particular niche. A fentanyl reservoir or matrix patch delivers opioid at an approximately constant rate for up to three days, avoiding first-pass metabolism and the peak-and-trough profile of oral dosing [5]. Buprenorphine patches serve a similar role in chronic non-malignant pain [6], and the lidocaine five per cent medicated plaster is included in international guidance for localised neuropathic pain [7]. Patch technology has advanced considerably in polymer chemistry and in adhesive and matrix design [8,9], yet the set of drugs available in a transdermal form remains small. The reason is pharmaceutical rather than technological: only molecules that cross the stratum corneum at a sufficient rate can be delivered through intact skin at a therapeutic dose.

The stratum corneum is a lipid-rich, highly organised barrier whose intercellular lamellae constitute the principal resistance to permeation [10,11,12]. The steady-state permeability coefficient Kp, with dimensions of length per unit time, summarises that resistance for a given solute and vehicle. Measuring Kp requires in vitro permeation testing on excised human skin, which is costly, limited by tissue availability and subject to considerable inter-donor and inter-laboratory variation. Predicting Kp from molecular structure has therefore been an objective of dermal pharmaceutics for more than three decades.

The reference model remains the equation of Potts and Guy, in which log Kp is a linear function of the octanol–water partition coefficient and molecular weight [13]. Numerous quantitative structure–permeability relationships have followed, using larger descriptor sets, non-linear regression, Gaussian processes, neural networks and, more recently, deep learning [14,15,16,17,18,19]. Two developments have changed what is possible. First, curated public databases of human skin permeation now exist: HuskinDB assembles Kp values measured on human skin together with the experimental protocol under which each was obtained [20], and SkinPiX extends the record with a systematic review of studies published between 2012 and 2021 [21]. Second, these resources have begun to be used for modelling; Waters and Quah fitted a multiple linear regression to HuskinDB and reported R^2^ = 0.85 [22], and a related study derived fragment contribution models from the same source [23]. Independently of the present work, Asgarkhanova et al. combined the same two databases into a quantitative structure–property model validated against three newly measured compounds, framed explicitly as a decision-support tool for pharmaceutical, cosmetic and occupational-safety applications [24]. That study reduces every compound with repeated measurements to a single consensus permeability value before fitting; the analysis reported here instead retains all 732 individual records, because the disagreement between repeated measurements of the same compound is itself the principal object of the present enquiry.

That reported performance, however, was obtained after severe curation. Of 253 compounds in HuskinDB, only 36 satisfied the four experimental criteria the authors imposed, and the remainder were discarded [22]. This raises a question that has not been addressed. If the whole of the available public data is retained, how well can skin permeability be predicted, and where does the limit lie? The question matters practically for transdermal analgesia, because the drugs of interest are a chemically diverse group spanning opioids, non-steroidal anti-inflammatory drugs and local anaesthetics, and because even for an established patch drug such as fentanyl, skin penetration varies markedly between and within individuals [25].

The present study therefore pursued three aims: to construct a quantitative structure–permeability model from the pooled public human dataset without protocol-based exclusion, and in doing so to characterise the functional form of the underlying structure–permeability relationship rather than assume the linear form of the reference equation; to test whether such a model extrapolates to analgesics treated as a held-out therapeutic class; and to determine what, if anything, still limits predictive accuracy once that functional form is captured.

## 2. Materials and Methods

### 2.1. Data Sources

Two open datasets were retrieved on 20 August 2026. HuskinDB was obtained from its public web service and contained 550 log Kp values for 253 compounds, released under a Creative Commons Attribution 4.0 licence [20]. SkinPiX version 1.1 was obtained from Recherche Data Gouv under the Etalab 2.0 open licence and contained 202 records, of which 201 carried a permeability coefficient, for 110 compounds measured on human skin only [21]. A third dataset, an updated compilation of maximum fluxes and permeability coefficients for compounds applied as aqueous solutions, was used solely to supply experimental octanol–water partition coefficients for a sensitivity analysis and was not used for model fitting.

Permeability coefficients were harmonised to log Kp in centimetres per second. Where a source reported values in centimetres per hour, log10(3600) was subtracted.

### 2.2. Structure Standardisation and Descriptors

Structures were parsed from the SMILES strings supplied by each database. Each structure was cleaned, reduced to its largest covalent fragment, neutralised, and stripped of isotope labels, so that salts such as lidocaine hydrochloride monohydrate were resolved to the parent drug [26,27]. Five carbon-free solutes, namely water, tritiated water, boric acid, borax and disodium octaborate tetrahydrate, were excluded because atom-contribution descriptors are not parameterised for them. Two substances that appeared under a single name with and without stereochemical annotation, codeine and nicotine, were unified onto the structure carrying the larger number of records.

Fifteen two-dimensional descriptors were computed with RDKit version 2026.03.5 [28]: molecular weight, Crippen log P and molar refractivity [29], topological polar surface area, counts of hydrogen bond donors and acceptors, rotatable bonds, aromatic rings, total rings, heavy atoms and heteroatoms, the fraction of sp3-hybridised carbon, the Labute approximate surface area, the Balaban J index and the Bertz complexity index; several of these descriptors are collinear (Figure S3), which is why the analysis below relies on permutation importance rather than on model coefficients.

Two analysis tables were derived. The compound-level table carried one row per standardised structure with log Kp taken as the median across all records for that compound, and was used for model fitting. The record-level table retained every individual measurement with its experimental metadata and was used for the protocol analysis.

### 2.3. Definition of the Analgesic Subgroup

Compounds were assigned to the analgesic subgroup if their name matched a term list drawn from Anatomical Therapeutic Chemical classes N02A, N02B, M01A and N01B. Opioid antagonists and papaverine were excluded because they are not analgesics. Drugs are named throughout by recommended international non-proprietary name; the source databases record pethidine as meperidine, indometacin as indomethacin and aminophenazone as aminopyrine.

### 2.4. Models and Validation

Six predictors were compared. The first applied the published Potts–Guy coefficients unchanged, with no fitting. The second refitted the same two-descriptor functional form to the present data. The remaining four were multiple linear regression on all fifteen descriptors, ridge regression with the penalty selected by internal cross-validation, a random forest of 800 trees, and gradient boosting with 700 trees, learning rate 0.03 and maximum depth four [30,31].

Compounds were split 80:20 into a training and a held-out test set, stratified jointly by log Kp quartile and analgesic status, with a fixed random seed. Hyperparameters were not tuned. The ensemble settings were fixed a priori at conventional defaults for datasets of this size (800 trees with a minimum leaf size of two for the random forest; 700 trees, learning rate 0.03 and maximum depth four for gradient boosting) and were not varied; the only quantity selected from data was the ridge penalty, chosen by internal cross-validation within the training set alone. The held-out test set took no part in fitting, in penalty selection or in any choice of setting, and was evaluated once. Internal performance was estimated by ten-fold cross-validation repeated five times within the training set; held-out performance was measured once on the held-out test set. Extrapolation to a new therapeutic class was assessed separately by refitting every model on the 287 non-analgesic compounds and predicting the 40 analgesics, a design that removes the class entirely from training [32,33].

The applicability domain was defined as the mean Euclidean distance, in standardised descriptor space, to the five nearest training compounds, with the threshold set at the 95th percentile of the corresponding distances within the training set [34,35].

To characterise the functional form of the relationship between the best-performing model and its descriptors, permutation importance was computed on the held-out test set as the mean decrease in the coefficient of determination over 50 random permutations of each descriptor, and one- and two-dimensional partial dependence was computed for the descriptors of highest importance.

### 2.5. Protocol Analysis

To separate chemical from experimental contributions, three record-level random forest models were fitted: on molecular descriptors alone, on protocol variables alone, and on both. Protocol variables were skin layer, anatomical site, diffusion cell type, donor and acceptor temperature and donor and acceptor pH; the spread of these variables across the pooled records is shown in Figure S1. Because several compounds contribute multiple records, all record-level cross-validation used grouped ten-fold splits in which every record for a given compound fell in the same fold, so that no compound appeared in both training and validation. A parallel analysis stratified compounds by skin layer and fitted models within each stratum.

### 2.6. Statistics

Paired comparisons of absolute prediction error used the Wilcoxon signed-rank test; unpaired comparisons of fold-wise R^2^ used the Mann–Whitney U test; association between computed and experimental log P used the Pearson correlation coefficient. *p* values are two-sided and given to full precision rather than thresholded. The computational pipeline ran on Python (version 3.11.10), drawing on scikit-learn (1.9.0), XGBoost (3.2.0) and SciPy (1.17.1) for model fitting and inference.

## 3. Results

### 3.1. Composition of the Pooled Dataset

After harmonisation, standardisation and exclusion, the analysis set comprised 732 permeability records for 327 compounds (Table 1). Median log Kp was −6.28 cm s^−1^ (interquartile range −7.30 to −5.38; full range −10.88 to −3.43). The compounds spanned a wide chemical space, with molecular weight from 32.0 to 765.0 g mol^−1^ (median 198.2) and Crippen log P from −7.57 to 6.92 (median 1.70). Twenty-nine compounds were common to both source databases, and 79 were contributed by SkinPiX alone, confirming that the two resources are complementary rather than redundant.

Forty compounds met the analgesic definition, comprising 12 opioids, 23 non-steroidal anti-inflammatory and other non-opioid analgesics and five local anaesthetics, and accounting for 96 records. The subgroup included the drugs actually marketed as transdermal analgesic systems, fentanyl with 15 records, sufentanil with 11 and lidocaine with eight. Buprenorphine was absent from both databases.

### 3.2. Variability Between Independent Studies

One hundred and fifty compounds carried more than one permeability record, and for 82 of them two or more independent source studies had reported a value. Among those 82, the median standard deviation of log Kp across studies was 0.54 log units (interquartile range 0.15 to 1.08), and the median spread between the highest and lowest reported value was 1.12 log units (interquartile range 0.38 to 2.83), with a maximum of 7.30. This dispersion arises between laboratories for a fixed chemical structure. It is an empirical estimate of the variability present in the pooled literature rather than a theoretical lower bound on model error, and it reflects differences in skin preparation, donor, vehicle, temperature, receptor pH and anatomical site as well as measurement reproducibility. A mixed-effects estimate of the same quantity is given below.

### 3.3. The Reference Equation Does Not Transfer

**Figure 1:**
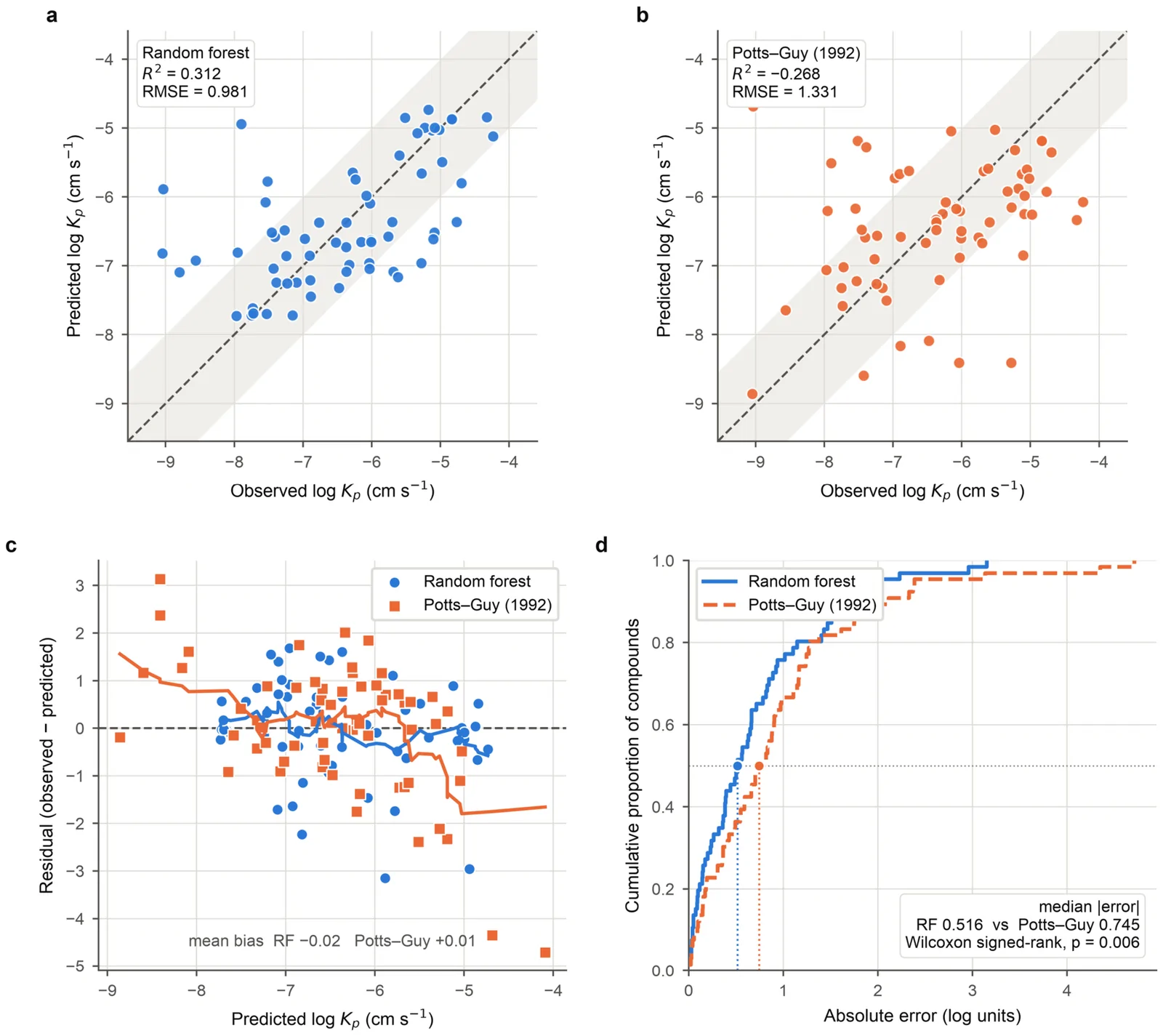
Behaviour of the fitted model and of the reference equation on the held-out test set of 66 compounds. (**a**) Observed against predicted log Kp for the random forest trained on the 261 training compounds. (**b**) The same compounds predicted by the published Potts–Guy equation [13] applied without refitting. In (**a**,**b**) the broken line is the line of identity and the shaded band marks agreement within one log unit. (**c**) Residuals against predicted values for both models; the heavy curves are running means. The annotated mean biases are near zero for both models, which is the point: the reference equation is conditionally biased, over-predicting permeable compounds and under-predicting poorly permeable ones, while averaging to almost no bias overall. The divergence of the two running-mean curves is the most direct evidence for the structural failure described in the text. (**d**) Empirical cumulative distribution of absolute error for both models, with the medians marked by dotted lines and circles. The Wilcoxon test quoted in the text compares medians; the curves separate over the lower and middle range of the error distribution and converge, crossing, in the upper tail, so the separation is not maintained across the whole range. All axes are in log Kp expressed in centimetres per second.

Applying the published Potts–Guy coefficients without refitting produced predictions worse than the mean of the observations, with R^2^ = −0.172 across all 327 pooled compounds and a root mean square error of 1.338. Performance on the held-out test set of 66 compounds was similar (R^2^ = −0.268, root mean square error 1.331). Figure 1a,b contrast the fitted random forest with the unmodified reference equation on the held-out test set. The failure is structural rather than random: the residuals of the reference equation trend downwards across the range of predicted values, so that permeable compounds are systematically over-predicted and poorly permeable compounds under-predicted, whereas the residuals of the random forest are approximately flat (Figure 1c). The cumulative distributions of absolute error (Figure 1d) separate over the lower and middle range rather than only at the median, although they converge and cross in the upper tail.

### 3.4. Model Comparison

Cross-validated and held-out performance for all six predictors is given in Table 2; observed-against-predicted plots for the four models omitted from Figure 1 are given in Figure S4. Within the training set the random forest achieved the highest cross-validated R^2^ at 0.375 (standard deviation 0.159) with root mean square error 0.948, followed by gradient boosting at 0.337 (standard deviation 0.199). Linear models were markedly weaker, the refitted Potts–Guy form reaching R^2^ = 0.238 and multiple linear regression on all descriptors only 0.172.

On the held-out test set of 66 compounds the ordering was preserved. The random forest gave R^2^ = 0.312, root mean square error 0.981 and mean absolute error 0.703; the median absolute error was 0.516 log units, against 0.745 for the unmodified reference equation, a difference that was statistically significant (Wilcoxon signed-rank W = 673.5, *p* = 0.006).

### 3.5. A Saturating, Non-Linear Structure–Permeability Relationship

**Figure 2:**
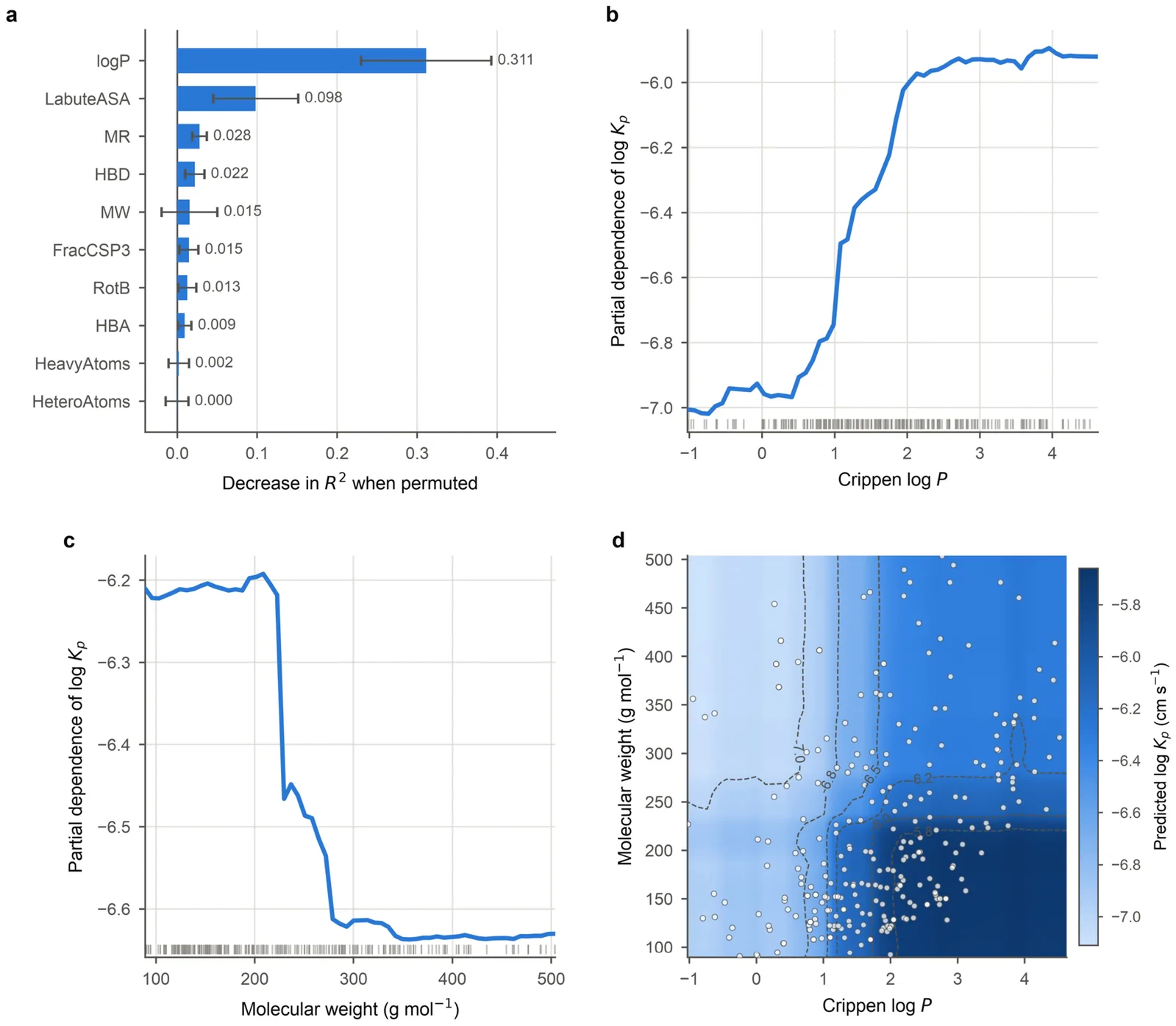
Structural determinants of predicted permeability in the random forest. (**a**) Permutation importance of the ten highest-ranked descriptors, measured on the held-out test set as the mean decrease in the coefficient of determination over 50 permutations; bars show the mean and whiskers one standard deviation. (**b**,**c**) Partial dependence of predicted log Kp on Crippen log P and on molecular weight; the tick marks along the lower axis show the distribution of the training compounds. (**d**) Joint partial dependence over the log P and molecular weight plane, with contours of equal predicted log Kp and open circles marking the compounds of the dataset.

Permutation importance on the held-out test set (Figure 2a) showed that predictive information is concentrated in lipophilicity and size. Crippen log P dominated with a mean decrease in R^2^ of 0.311 (standard deviation 0.081), followed by the Labute approximate surface area at 0.098 and molar refractivity at 0.028; the remaining descriptors contributed little.

**Figure 3:**
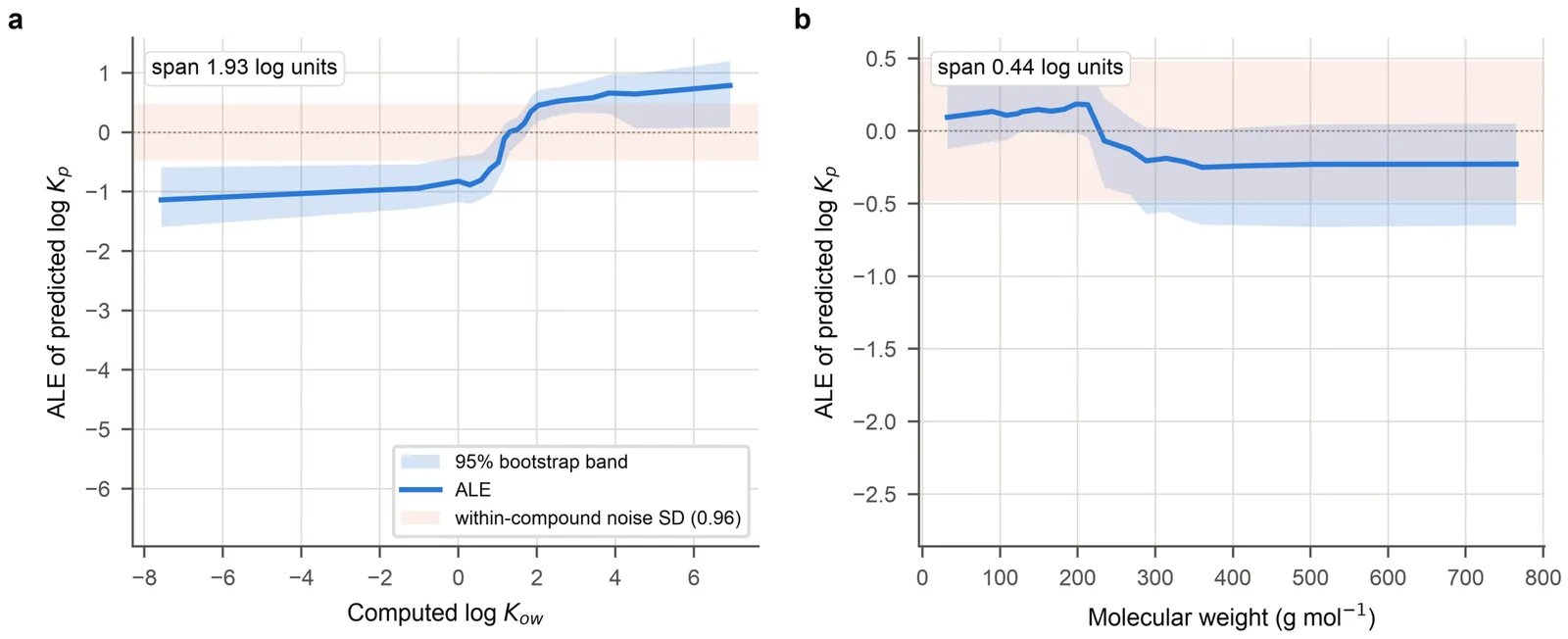
Accumulated local effects for the two descriptors that carry the mechanistic claims, with 95 per cent bands from 200 bootstrap refits of the forest. Accumulated local effects are used in preference to partial dependence because the descriptors are collinear (Figure S3) and partial dependence evaluates the model at descriptor combinations the data do not contain. The shaded horizontal band marks the within-compound standard deviation estimated from the mixed-effects model (0.963 log units), so the vertical travel of each curve can be read against the variability between repeated measurements of the same compound. (**a**) Crippen log P. (**b**) Molecular weight.

The form of these dependences is instructive (Figure 2b,c). Predicted log Kp rose by 1.12 log units across the range of log P, but not linearly: the increase was concentrated near log P = 1.2 and the curve plateaued above log P = 2.5, so that further lipophilicity bought no further permeability. Molecular weight acted over a smaller range of 0.44 log units, with predicted permeability falling near 220 g mol^−1^. Because partial dependence is unreliable when descriptors are collinear, and molecular weight correlates with the Labute approximate surface area at Spearman rho = 0.97 (Figure S3), both dependences were recomputed as accumulated local effects with bootstrap bands (Figure 3). The lipophilicity saturation survived that check, spanning 1.93 log units against a within-compound noise standard deviation of 0.963. The molecular-weight effect did not: its span of 0.44 log units was smaller than the mean width of its own 95 per cent bootstrap band (0.54), so the apparent threshold is a feature of the fitted model rather than a relationship the data resolve. Consistent with this, molecular weight carried no information independent of the Labute surface area (partial r = 0.008, *p* = 0.88 given that descriptor), and removing it from the descriptor set changed cross-validated performance from R^2^ = 0.364 to 0.366. The joint surface (Figure 2d) shows the consequence: contours of equal predicted permeability are neither parallel nor evenly spaced, which is precisely the behaviour a bilinear equation such as Potts–Guy cannot represent.

### 3.6. Extrapolation to Analgesics

When all 40 analgesics were withheld from training, every model retained some predictive ability, but the ranking inverted. The refitted two-descriptor linear model performed best, with R^2^ = 0.361 and root mean square error 0.781, followed by ridge regression at R^2^ = 0.318. The random forest fell to R^2^ = 0.108 and gradient boosting to R^2^ = −0.201. The median absolute error of the two-descriptor model, 0.568 log units, was significantly smaller than that of the unmodified Potts–Guy equation at 0.794 (W = 247.0, *p* = 0.029), but the advantage over the random forest at 0.610 was not statistically significant (W = 353.0, *p* = 0.444).

**Figure 4:**
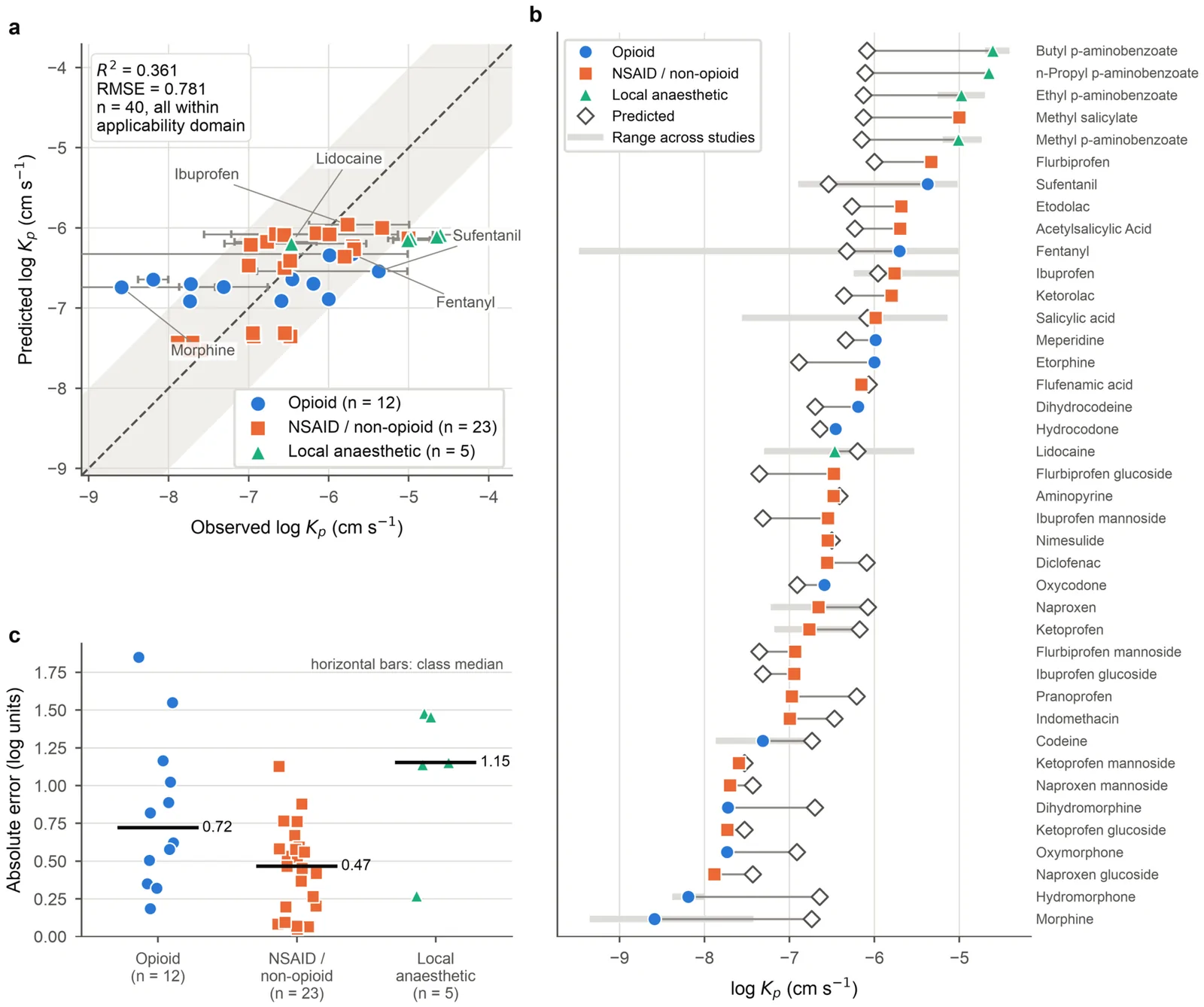
Prediction of the analgesic subgroup when the entire class is withheld from training, using the two-descriptor linear model fitted to the 287 non-analgesic compounds. (**a**) Observed against predicted log Kp; horizontal whiskers give the range of values reported for compounds measured more than once, the broken line is the line of identity and the shaded band marks agreement within one log unit. Five drugs of particular relevance to transdermal delivery are labelled. (**b**) The same 40 drugs ranked by observed log Kp; filled symbols are observed values, open diamonds are predictions, and the grey bar behind each row is the range reported across studies. (**c**) Absolute prediction error by pharmacological class, with a horizontal bar at each class median.

Figure 4 shows these leave-analgesics-out predictions. Errors were not uniform across the subgroup. Ranking the 40 drugs by observed permeability (Figure 4b) shows that the model compressed the range at both extremes, over-predicting the least permeable opioids such as morphine and under-predicting the most permeable compounds, including fentanyl and sufentanil. By class (Figure 4c) the median absolute error was smallest for the non-steroidal anti-inflammatory and other non-opioid analgesics at 0.47 log units, intermediate for the opioids at 0.72, and largest for the five local anaesthetics at 1.15, although the last estimate rests on few compounds. All 40 analgesics lay within the applicability domain of the training set (Figure S5), so these errors reflect model bias rather than extrapolation beyond the chemical space sampled.

### 3.7. Where the Limit Lies

Even after this non-linear relationship is captured, three lines of evidence indicate that residual accuracy is limited by experimental heterogeneity rather than by further refinement of molecular description.

**Figure 5:**
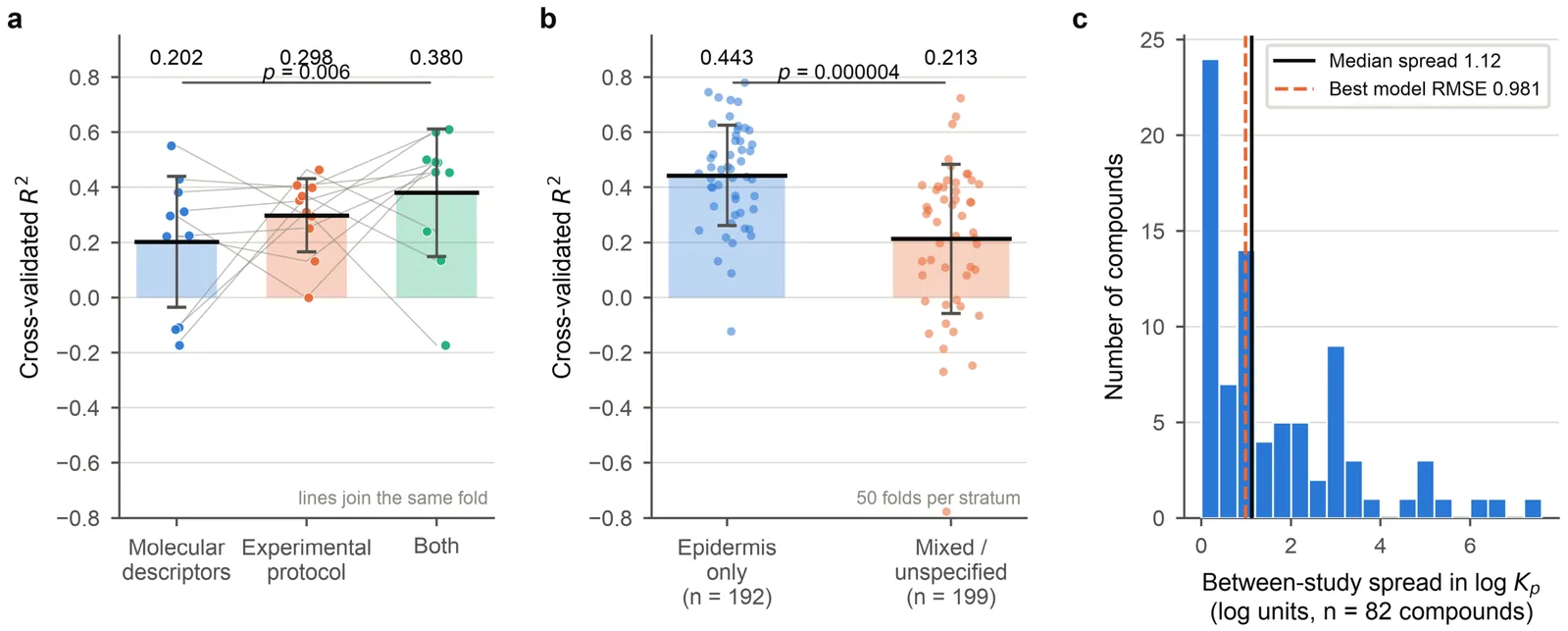
Sources of the limit on predictive accuracy. (**a**) Cross-validated coefficients of determination for record-level random forest models fitted to molecular descriptors alone, to experimental protocol variables alone, and to both, using grouped cross-validation by compound; the three models were scored on the same ten folds, and the faint lines join each fold across the three conditions, so that the frequent crossings between the first two conditions correspond to the non-significant paired comparison reported in the text. (**b**) Compound-level models fitted separately within the epidermis stratum and within the mixed or unspecified stratum, each over 50 repeated cross-validation folds. In (**a**,**b**) the individual points are the per-fold values, the heavy horizontal line is their mean, whiskers show one standard deviation, the bracket gives the exact *p* value for the comparison indicated, and both panels share one vertical scale. (**c**) Distribution of the spread in log Kp between independent studies for the 82 compounds measured more than once; the solid line marks the median spread and the broken line the root mean square error of the best model.

First, at the record level with grouped cross-validation, molecular descriptors alone gave R^2^ = 0.202, protocol variables alone gave R^2^ = 0.298, and the two together gave R^2^ = 0.380 (Figure 5a). Cross-validation folds share training data and are not independent samples, so these comparisons are reported with the Nadeau–Bengio correction for resampled tests alongside the effect sizes. The combined model was better than chemistry alone by 0.178 in R^2^ (95 per cent interval 0.101 to 0.249; corrected *p* = 0.014). Protocol alone exceeded chemistry alone by 0.096, but the interval spans zero (−0.076 to 0.285; corrected *p* = 0.520), so the two information sources are of comparable value and cannot be ranked on these data.

Second, restricting the analysis to compounds measured on epidermis preparations raised mean cross-validated R^2^ from 0.213 to 0.443 (Figure 5b), a difference of 0.231 with a standardised effect size of 0.99. This comparison needs three qualifications. Treating the 50 repeated folds in each stratum as independent samples overstates the evidence; with the Nadeau–Bengio correction the difference is no longer significant at conventional levels (*p* = 0.059). A permutation test that reassigned compounds to two pseudo-strata of the same sizes, preserving each compound’s own data and moving only its label, likewise placed the observed gap inside the null distribution (two-sided *p* = 0.184). That null distribution is shown in Figure S8a. The gap is not, however, explained by a difference in chemical space: the fifteen descriptors were already balanced between the strata before matching, and after nearest-neighbour matching on standardised descriptors the gap persisted at 0.203. Balance before and after matching is shown in Figure S8b. The strata also overlap, since 84 compounds were measured on both kinds of preparation, so this is not a clean contrast between independent sets of compounds. The epidermis stratum contained 192 compounds, more than five times the number retained by the most closely comparable published curation. We therefore report skin layer as a large but statistically unestablished effect that survives a chemical-space control.

Third, the failure of the reference equation is not attributable to the use of a calculated rather than a measured partition coefficient. For the 98 compounds with an experimental octanol–water value, computed and experimental log P agreed closely (Pearson r = 0.853, *p* = 8.39 × 10^−29^, mean absolute difference 0.62; Figure S2). Substituting the experimental value into the Potts–Guy equation left the coefficient of determination negative, improving it only from −0.218 to −0.085, and the reduction in absolute error was not significant (W = 1993.0, *p* = 0.125).

Taken together with the dispersion of the reference measurements themselves, these results place the model close to the accuracy the data can support. For 56 per cent of the 82 compounds measured in more than one study, the disagreement between studies exceeded the root mean square error of the best model, and the median between-study spread of 1.12 log units is of the same order as that error of 0.981 (Figure 5c).

### 3.8. Sensitivity of the Results to Analysis Choices

Four checks were run on the choices that most directly affect the headline numbers; full tables are given in the Supplementary Materials.

Collapsing each compound to a median log Kp is not what drives the results. Repeating the whole analysis with the mean in place of the median changed 63 of 327 compound values, by at most 1.15 log units, and moved every performance figure by 0.01 or less. Fitting at the record level instead, with grouped cross-validation and a held-out split defined on compounds throughout so that no compound was ever divided between folds, lowered cross-validated performance substantially (random forest R^2^ = 0.203 against 0.364) while raising held-out performance slightly (0.336 against 0.312). The three treatments are set side by side for all six models in Figure S6a–c. The fall is expected: a record-level model is asked to reproduce the scatter between repeated measurements as well as the difference between compounds. The ordering of the two leading models was unchanged under all three treatments, although the weaker models exchanged places.

A mixed-effects model with compound as a random intercept separates those two sources directly. Between-compound variance was 0.862 and within-compound variance 0.928, so 52 per cent of the record-level variance lies between repeated measurements of the same compound, and the within-compound standard deviation of 0.963 log units is close to the 0.981 root mean square error of the best model. Adding the fifteen descriptors as fixed effects explained 46 to 48 per cent of the between-compound variance and left the within-compound variance essentially untouched (0.928 to 0.931), which is what a structure-based description should be expected to do. The variance components are shown in Figure S6d.

Because performance on a single 80:20 split is an unstable quantity at this sample size, the split was repeated 200 times with the same stratification. The random forest had a median held-out R^2^ of 0.383 with a 95 per cent interval of 0.144 to 0.559, took first place in 79 per cent of splits and beat gradient boosting in 89 per cent; the single split reported in Table 2 sits at the 26th percentile of that distribution, so it is not a favourable draw. The distribution, and the position of the reported split within it, are shown in Figure S7. The three weaker fitted models were separated by less than 0.02 in median R^2^ and cannot be ranked against one another on these data.

Finally, ionisation was computed rather than assumed. Using the site pKa library of Dimorphite-DL (version 2.0.2), 107 of 327 compounds carried an ionisable group at pH 7.4 (69 basic, 23 acidic, 15 ampholytic) and the median fraction ionised was zero. The composition of the ionisable set is given in Table S6. Adding the fraction ionised, and its anionic and cationic components, to the descriptor set changed cross-validated performance from R^2^ = 0.364 to 0.367 for the random forest and from 0.223 to 0.228 for the two-descriptor model. On these data the limit is therefore not a missing account of ionisation.

## 4. Discussion

This study shows that, across the whole of the openly available human skin permeability data, log Kp rises with lipophilicity in a saturating rather than linear fashion, a form that the bilinear Potts–Guy equation cannot represent. The model also learned a decline in permeability above a molecular weight near 220 g mol^−1^, but that effect is not resolved by these data and is reported as model-derived rather than as a property of skin. A model that captures this form reaches a coefficient of determination of about 0.31 on unseen compounds, and the accuracy that remains achievable is limited predominantly by heterogeneity of the experiments from which the data were assembled rather than by further refinement of molecular description.

This saturating dependence on lipophilicity is consistent with a partition-limited mechanism of transport across the lipid-rich stratum corneum: once a solute is lipophilic enough to partition favourably into the intercellular lamellae, further lipophilicity adds little to the rate at which it then diffuses across the barrier [11,12]. A size-dependent restriction on diffusion through that same structure would be the natural counterpart for molecular weight, and it is the reading an earlier version of this work adopted; the present data do not resolve an effect of that size, and we therefore do not argue for one. Because the Potts–Guy equation assumes a constant marginal effect of lipophilicity across its full range, it necessarily misrepresents compounds on either side of the point at which that dependence saturates, which is the structural reason, visible directly in the residual pattern of Figure 1c, that its predictions deteriorate systematically rather than randomly.

This result should be read alongside, not against, the higher figures reported previously. Waters and Quah obtained R^2^ = 0.85 from the same primary source, but did so after retaining 36 of 253 compounds that shared an anatomical site, skin layer, donor concentration and temperature [22]; the fragment contribution study that followed applied the same condition-based filtering and reported R^2^ = 0.71 in a training set of 29 compounds and 0.89 in a test set of seven [23]. The present analysis reproduces the underlying phenomenon in a form that separates it from the choice of model: within a single skin layer, performance approximately doubles, and it does so on 192 compounds rather than a few dozen. What earlier work achieved by discarding data, and thereby narrowing the applicability of the resulting equation, is here shown to be a property of the data themselves.

A contemporaneous study by Asgarkhanova et al., built on the same two databases, illustrates a different way of using the same raw material [24]. That work assembles a quantitative structure–property model from a single consensus Kp value per compound, draws on the originating laboratory’s own fragment-based descriptor framework to judge from its cited methodological sources, and validates the model against three newly measured compounds with the stated aim of supporting decision-making in pharmaceutical, cosmetic and occupational-safety contexts. We have not been able to obtain the full text of that article and therefore make no claim about what it reports in its results or discussion; the comparison offered here is limited to what its abstract, deposited data and reference list state. On that basis, the two studies are complementary rather than competing: the present analysis retains all 732 individual records specifically so that the between-study dispersion documented above can be measured and related to model error, incorporates a third, independent dataset to test whether a measured rather than a computed partition coefficient changes the picture, and extends validation to a chemically defined class, transdermal analgesics, under a pre-specified leave-class-out design with paired significance testing. Whether a consensus value calculated before modelling absorbs part of the variance identified here as a ceiling on accuracy is a question the present data cannot settle, but the scale of that variance, a median between-study spread of 1.12 log units, suggests it is worth asking directly.

The failure of the unmodified Potts–Guy equation deserves comment, since it remains the most widely applied predictor in dermal risk assessment and formulation screening. The equation was derived from a compilation dominated by simple, mostly small and neutral solutes measured under comparatively uniform conditions [13,36], a chemical range narrow enough that a linear approximation of the underlying relationship performs adequately. The pooled modern dataset spans a far wider range of lipophilicity and molecular weight, including ionisable drugs, prodrug conjugates and large lipophiles, over which the relationship visibly departs from linearity (Figure 2b–d). The demonstration that substituting experimental for computed log P does not rescue performance confirms that the shortfall is one of functional form and experimental transferability rather than of descriptor quality.

Several specific protocol factors are known to shift measured permeability. Skin layer determines whether the dermis contributes an additional aqueous diffusional resistance, which disproportionately affects lipophilic solutes. Temperature alters both the fluidity of stratum corneum lipids and the diffusion coefficient, and heating has been shown experimentally to increase percutaneous absorption of model penetrants [37]. Receptor fluid pH governs the ionisation state of weak acids and bases at the membrane interface and thereby their effective partitioning [38,39]. Analyses of the EDETOX compilation reached a comparable conclusion, namely that formulation and experimental conditions account for a substantial share of the variance in in vitro human skin permeation [40], and modelling of in vitro permeation testing variability continues to be an active concern [41]. Our finding that protocol variables carry predictive signal comparable to that of molecular descriptors is consistent with that literature.

That comparison must nevertheless be stated carefully. Protocol and chemistry are confounded in these data, because investigators do not choose experimental conditions at random with respect to the compounds they study: work on stratum corneum sheets tends to involve different solutes from work on full-thickness skin, and particular laboratories study particular chemical series. The correct inference is that protocol descriptors carry predictive information of comparable magnitude to molecular descriptors, not that protocol is more important than chemistry. Our own statistical test is explicit on this point, the difference between the two being far from significant.

The analgesic analysis has a practical bearing on transdermal product development. Withholding an entire therapeutic class from training is a demanding test, and it is notable that the simplest model, a two-descriptor linear equation refitted to the present data, extrapolated at least as well as the ensemble methods and significantly better than the original Potts–Guy coefficients. Tree-based models interpolate well within the region of descriptor space they have seen and cannot extend a trend beyond it; where the intended use is to screen a new chemical class, a parsimonious and monotone model may be preferable despite lower apparent accuracy. That all 40 analgesics fell inside the applicability domain is reassuring for the use of such models in early screening, but it should not be read on its own. Predictions were systematically compressed at both extremes, and the drugs that sit at those extremes are the clinically important ones: the least permeable opioids such as morphine are over-predicted and the most permeable, fentanyl and sufentanil, are under-predicted. A model of this accuracy can rank candidate analgesics approximately and cannot substitute for measurement when a delivery rate must be specified. The observed variation in fentanyl penetration between individuals underlines the same point [25].

This study has several limitations. The descriptors are two-dimensional, so the ten groups of stereoisomers present in the dataset, including the three hexabromocyclododecane diastereomers and the glucoside and mannoside conjugates of four non-steroidal anti-inflammatory drugs, are indistinguishable to the model although their measured permeabilities differ. Assignment to the analgesic subgroup was made by matching compound names to a term list rather than by formal Anatomical Therapeutic Chemical coding, and although the resulting list was inspected, misclassification of borderline agents cannot be excluded. Vehicle composition, which is known to modify permeability substantially, was not modelled because it is recorded inconsistently across the two sources. The applicability domain was defined by a single distance criterion, and alternative formulations may give different coverage [35]. Finally, the analysis rests on two databases; although they are the most complete openly licensed human resources currently available, both inherit whatever reporting and publication biases affect the primary literature.

The implication for the field is twofold. First, models intended for pooled, non-curated public data should allow the effect of lipophilicity to saturate rather than assume the bilinear form of the Potts–Guy equation; this alone recovers a materially better fit than the reference equation, without requiring further increases in model complexity. Second, beyond capturing that functional form, further increases in model complexity are unlikely to yield much when independent measurements of the same compound differ by a median of more than one log unit between laboratories, a dispersion of the same order as the total error of the best model reported here. Additional return should come from the reporting side: consistent specification of skin layer, thickness, anatomical site, donor and receptor composition, pH and temperature, as recommended for dermal absorption studies conducted under established test guidelines [42], and deposition of complete protocol metadata alongside every reported permeability coefficient. Both databases used here already capture much of this information, and the present analysis indicates how much is gained when it is complete.

## 5. Conclusions

Human skin permeability, analysed across the whole of the openly available public data (732 records, 327 compounds), depends on lipophilicity in a saturating rather than linear fashion, a form the widely used Potts–Guy equation cannot represent; the accompanying decline above a molecular weight near 220 g mol^−1^ is not resolved by these data and is reported as model-derived. A random forest that captures this form predicts log Kp for unseen compounds with a coefficient of determination of 0.312 and a root mean square error of 0.981 log units, significantly better than the unmodified Potts–Guy equation but far below figures reported from heavily curated subsets. Restricting the analysis to a single skin layer nearly doubles performance, and experimental protocol variables carry predictive information comparable to that of molecular descriptors. When an entire therapeutic class, 40 transdermal analgesics, was withheld from training, however, a simple linear model extrapolated better than the non-linear ensemble methods, all 40 falling within the applicability domain; capturing the correct functional form therefore improves interpolation within the sampled chemical space but is not sufficient on its own for extrapolation beyond it. Beyond functional form, the accuracy achievable from public data is limited at least as much by standardisation of the underlying experiments as by model sophistication; the two information sources could not be ranked against each other on these data (W = 21.0, *p* = 0.557), and improving the completeness of reported protocol metadata should take precedence over further algorithmic refinement.

## Figures and Tables

**Table 1:** Composition and physicochemical characteristics of the pooled human skin permeability dataset.

Characteristic	All Compounds (n = 327)	Analgesic Subgroup (n = 40)
Source databases		
HuskinDB: records/compounds	550/253	–
SkinPiX v1.1: records/compounds	201/110	–
Pooled after standardisation: records/compounds	732/327	96/40
Compounds shared by both databases	29	–
Compounds with >1 permeability record	150	14
Compounds reported by ≥2 independent studies	82	13
Between-study SD of log Kp, log units, median (IQR)	0.54 (0.15 to 1.08)	–
Between-study spread of log Kp, log units, median (IQR)	1.12 (0.38 to 2.83)	–
Pharmacological class		
Opioid analgesics (ATC N02A)	–	12
NSAID and other non-opioid analgesics (ATC M01A, N02B)	–	23
Local anaesthetics (ATC N01B)	–	5
Physicochemical characteristics, median (IQR) [range]		
log Kp (cm s^−1^)	−6.28 (−7.30 to −5.38) [−10.88 to −3.43]	−6.48 (−6.98 to −5.75) [−8.59 to −4.61]
Molecular weight (g mol^−1^)	198.2 (139.0 to 310.3) [32.0 to 765.0]	287.4 (233.6 to 360.5) [138.1 to 416.4]
Crippen log P	1.70 (0.86 to 2.67) [−7.57 to 6.92]	1.68 (1.05 to 3.05) [0.29 to 4.36]
Topological polar surface area (Å^2^)	44.8 (26.9 to 75.3) [0.0 to 288.5]	52.9 (45.4 to 68.9) [23.6 to 133.5]
Hydrogen bond donors	1 (0 to 2) [0 to 11]	1 (1 to 2) [0 to 4]
Hydrogen bond acceptors	3 (1 to 4) [0 to 16]	3 (2 to 5) [1 to 8]
Rotatable bonds	2 (1 to 5) [0 to 29]	3 (1 to 5) [0 to 8]

ATC = Anatomical Therapeutic Chemical classification; IQR = interquartile range; Kp = permeability coefficient; NSAID = non-steroidal anti-inflammatory drug; SD = standard deviation; Å^2^ = square angstrom (1 Å^2^ = 10^−20^ m^2^). A dash indicates that the quantity does not apply. Buprenorphine is absent from both source databases.

**Table 2:** Predictive performance of six models for human skin permeability.

Model	CV R^2^ (SD)	CV RMSE	Held-Out R^2^	Held-Out RMSE	Held-Out MAE	LAO R^2^	LAO RMSE
Potts–Guy equation, published coefficients	Not fitted	Not fitted	−0.268 [−0.638, 0.199]	1.331	0.953	0.010	0.972
Potts–Guy functional form, refitted	0.238 (0.139)	1.060	0.078 [0.078, 0.377]	1.135	0.909	0.361	0.781
Multiple linear regression, all descriptors	0.172 (0.266)	1.093	0.066 [−0.011, 0.394]	1.143	0.894	0.133	0.910
Ridge regression, all descriptors	0.191 (0.159)	1.089	0.144 [0.062, 0.380]	1.094	0.885	0.318	0.807
Random forest	0.375 (0.159)	0.948	0.312 [0.144, 0.559]	0.981	0.703	0.108	0.923
Gradient boosting	0.337 (0.199)	0.970	0.274 [0.055, 0.531]	1.007	0.746	−0.201	1.071

CV = cross-validated (ten-fold, repeated five times, training set of 261 compounds); Held-Out = held-out test set (66 compounds); LAO = leave-analgesics-out (40 analgesics, refitted on the 287 non-analgesic compounds); MAE = mean absolute error; RMSE = root mean square error; SD = standard deviation. All values are in log Kp units (cm s^−1^). The published Potts–Guy coefficients were applied without fitting, so no cross-validation applies. Values in brackets after the held-out R^2^ are the 2.5th to 97.5th percentiles of that model’s held-out R^2^ over 200 repeated stratified splits, and describe the variability of the estimate rather than an interval around the single split reported.

## Data Availability

The data analysed in this study are third-party public datasets. HuskinDB is available from its public web service under a Creative Commons Attribution 4.0 licence [20]. SkinPiX version 1.1 is available from Recherche Data Gouv (https://doi.org/10.57745/7FHQOY) (accessed on 20 August 2026) under the Etalab 2.0 open licence [21]. No new data were generated. All analysis code and the pooled analysis-ready dataset, together with every derived table behind the figures, are publicly available at https://github.com/sanghyunn82-creator/skin-permeability-kp (accessed on 13 September 2026) under the MIT licence, and the exact version underlying this article is archived as the tagged release v1.0.0 at https://github.com/sanghyunn82-creator/skin-permeability-kp/releases/tag/v1.0.0 (accessed on 13 September 2026).

## Supplementary Materials

The following supporting information can be downloaded at: https://www.mdpi.com/article/10.3390/medicina62101929/s1, Figure S1: Diversity of experimental protocol among the 732 pooled records; Figure S2: Agreement between computed and experimentally measured octanol–water partition coefficient for the 98 compounds with a measured value available in the third dataset described in Materials and Methods; Figure S3: Spearman rank correlation between the fifteen two-dimensional descriptors used for model fitting; Figure S4: Observed against predicted log Kp on the held-out test set of 66 compounds for the four models omitted from Figure 1: (a) the Potts–Guy functional form refitted to the present data, (b) multiple linear regression on all fifteen descriptors, (c) ridge regression, and (d) gradient boosting; Figure S5: Applicability-domain distance, the mean Euclidean distance in standardised descriptor space to the five nearest training compounds, for the 40 analgesics withheld during leave-analgesics-out extrapolation, grouped by pharmacological class; Table S1: Performance of all six models under three treatments of repeated measurements of the same compound; Table S2: Variance components of a mixed-effects model of log Kp at record level, with compound as a random intercept; Table S3: Distribution of held-out performance across 200 repeated stratified 80:20 splits; Table S4: Comparisons between descriptor sets and between skin-layer subsets, corrected for the dependence between cross-validation folds; Table S5: Effect of adding computed ionisation descriptors at pH 7.4 to the descriptor set; Figure S6: Sensitivity of every reported figure to the treatment of repeated measurements of the same compound; Figure S7: Held-out performance across 200 repeated stratified 80:20 splits of the compounds; Figure S8: Whether the skin-layer difference is an artefact of chemical space; Table S6: Composition of the ionisable set at pH 7.4, computed with the site pKa library of Dimorphite-DL; Conformity with the OECD principles for the validation of (Q)SAR models.
