# Supplementary material for: Human Skin Permeability Prediction by Machine Learning for Transdermal Drug Delivery
Source: Medicina (Kaunas). 2026 Oct 7;62(10):1929. doi: 10.3390/medicina62101929 (PMC13706845; doi:10.3390/medicina62101929)
Supplement: Supplementary file 1 [file medicina-62-01929-s001.zip › medicina-4595038-supplementary.pdf]

Human Skin Permeability Prediction by Machine Learning for Transdermal Drug Delivery

Supplementary Materials

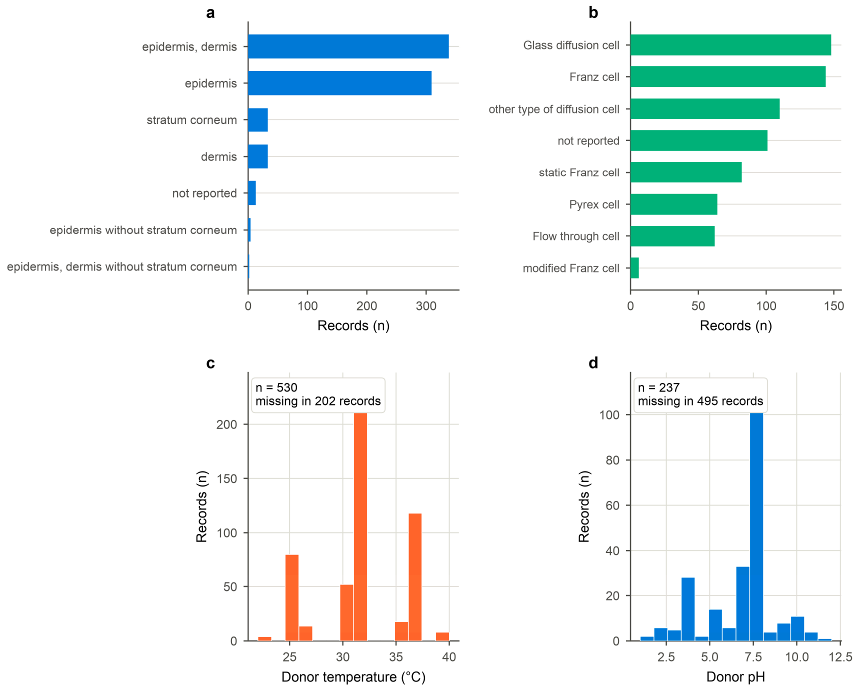

Figure S1. Diversity of experimental protocol among the 732 pooled records. (a) Skin-layer preparations. (b) Diffusion cell types. (c) Donor-compartment temperature (recorded for 530 of 732 records). (d) Donor-compartment pH (recorded for 237 of 732 records).

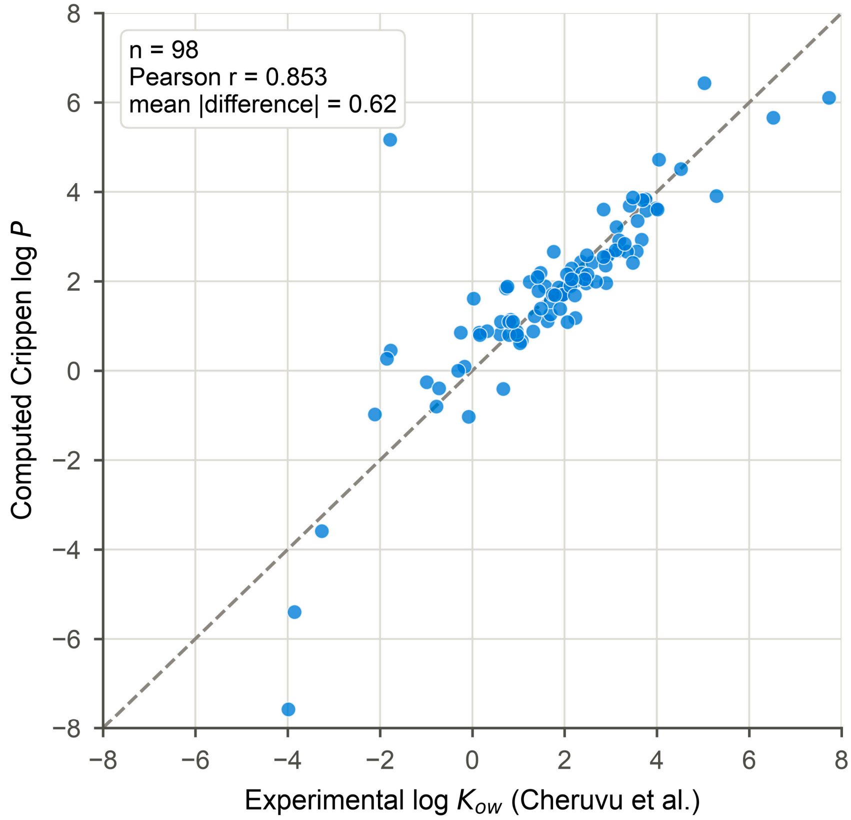

Figure S2. Agreement between computed and experimentally measured octanol–water partition coefficient for the 98 compounds with a measured value available in the third dataset described in Materials and Methods. The broken line is the line of identity.

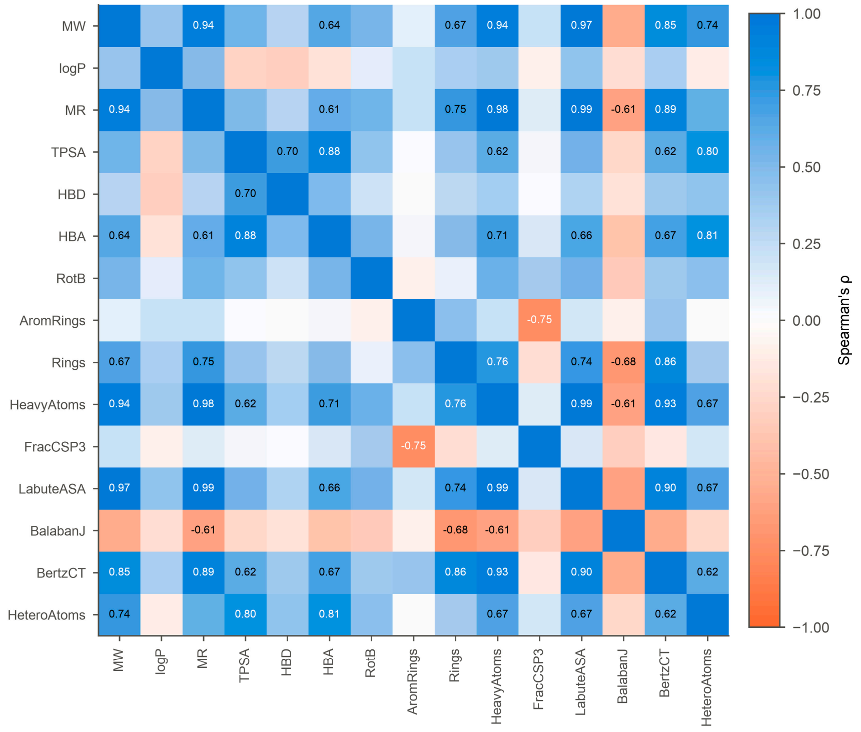

Figure S3. Spearman rank correlation between the fifteen two-dimensional descriptors used for model fitting. Coefficients are printed where the magnitude is 0.6 or greater.

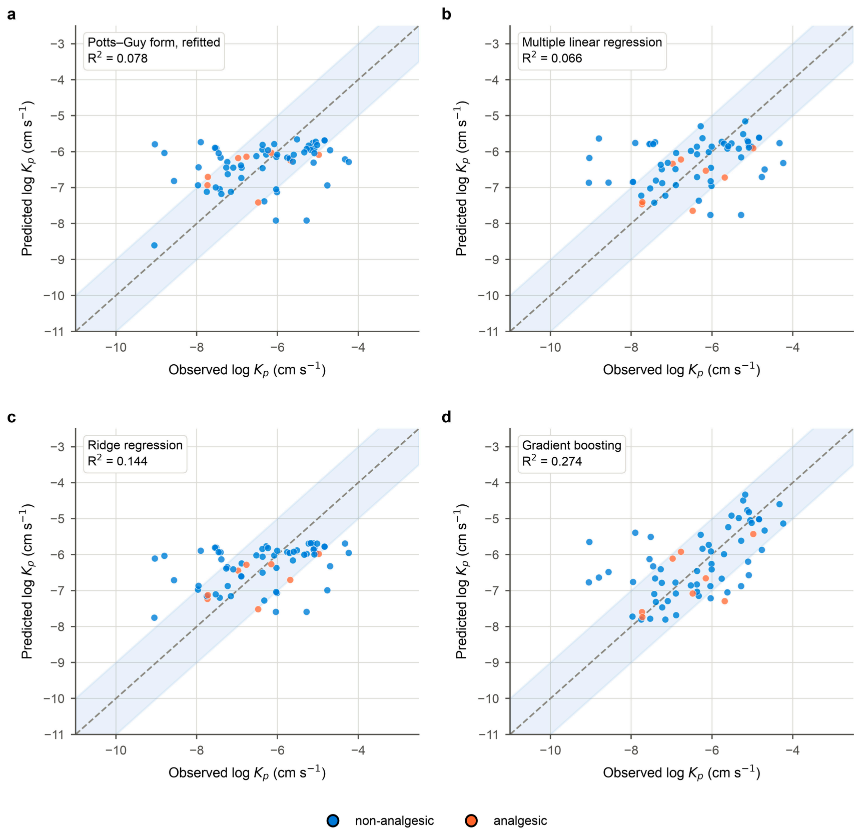

Figure S4. Observed against predicted log Kp on the held-out test set of 66 compounds for the four models omitted from Figure 1: (a) the Potts–Guy functional form refitted to the present data, (b) multiple linear regression on all fifteen descriptors, (c) ridge regression, and (d) gradient boosting. The broken line is the line of identity and the shaded band marks agreement within one log unit; colour distinguishes analgesic from non-analgesic compounds. Coefficients of determination match Table 2.

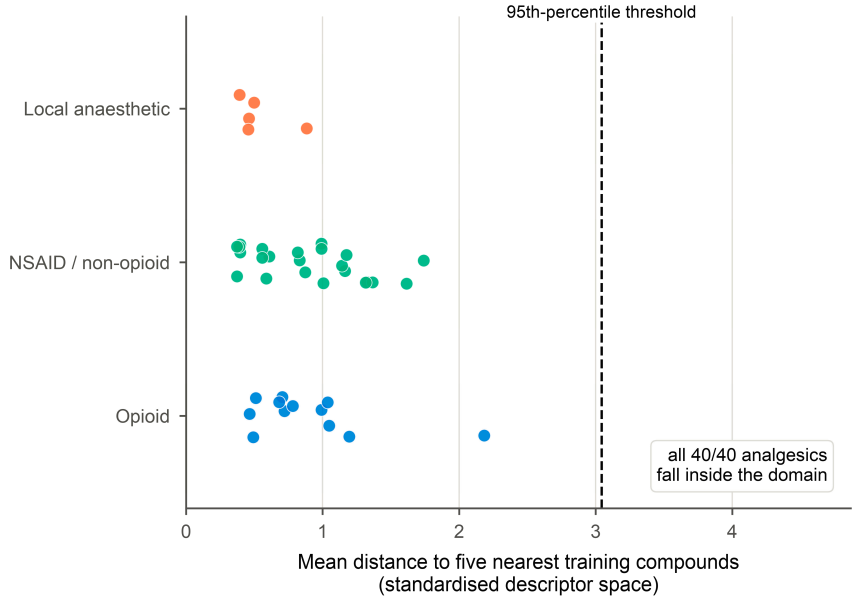

Figure S5. Applicability-domain distance, the mean Euclidean distance in standardised descriptor space to the five nearest training compounds, for the 40 analgesics withheld during leave-

analgesics-out extrapolation, grouped by pharmacological class. The broken line marks the 95th-percentile threshold that defines the applicability domain; all 40 compounds fall to its left.

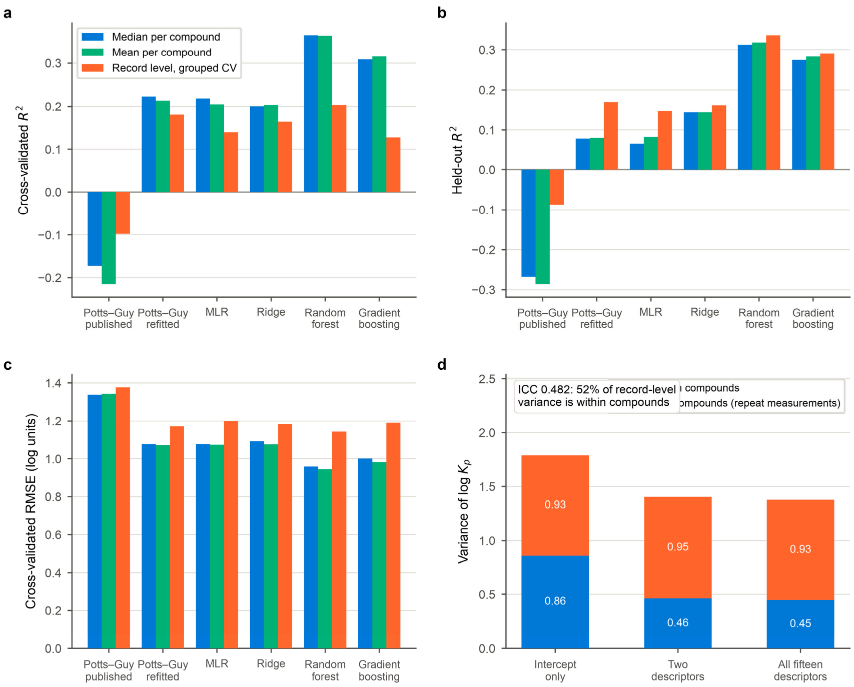

Figure S6. Sensitivity of every reported figure to the treatment of repeated measurements of the same compound. (a) Cross-validated R2, (b) held-out R2 and (c) cross-validated root mean square error for all six models under three treatments: the median per compound used throughout the manuscript, the mean per compound, and fitting at record level with cross-validation folds grouped by compound so that no compound is divided between folds. (d) Variance components of a mixed-effects model of log Kp at record level with compound as a random intercept, for three specifications of the fixed effects; the intraclass correlation of the intercept-only model places 52 per cent of the record-level variance within compounds, and adding descriptors reduces the between-compound component while leaving the within-compound component untouched.

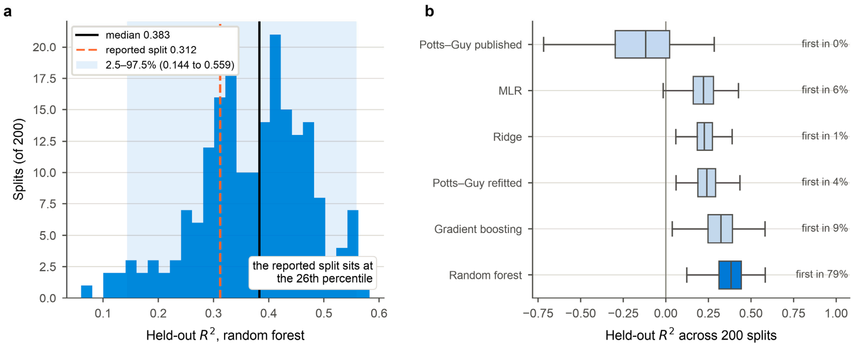

Figure S7. Held-out performance across 200 repeated stratified 80:20 splits of the compounds. (a) Distribution for the random forest, with the median, the 2.5th to 97.5th percentile range, and the position of the single split reported in Table 2. (b) Distribution for all six models, ordered by mean rank, with the percentage of splits in which each model had the highest held-out R2. The percentile range describes the variability of the estimate across splits and is not a confidence interval for any one split.

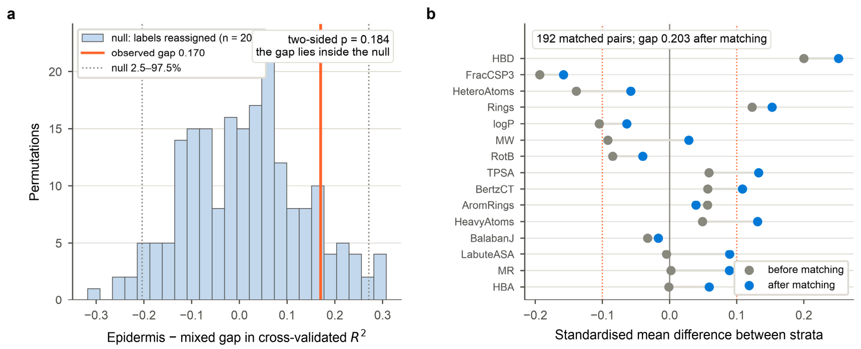

Figure S8. Whether the skin-layer difference is an artefact of chemical space. (a) Null distribution of the difference in cross-validated R2 between the two strata when compounds are reassigned to pseudo-strata of the same sizes, each compound keeping its own data and only its label moving; the observed difference lies inside that null (two-sided p = 0.184). (b) Standardised mean difference between the strata for each of the fifteen descriptors, before and after nearest-neighbour matching on standardised descriptors; dotted lines mark the conventional 0.1 threshold. Both panels use the reduced settings of the permutation analysis (150 trees, ten folds), so the observed difference differs slightly from the value in the main text.

Table S1. Performance of all six models under three treatments of repeated measurements of the same compound.

Model	Median per compound, CV R2	Median per compound, held-out R2	Mean per compound, CV R2	Mean per compound, held-out R2	Record level, grouped CV, CV R2	Record level, grouped CV, held-out R2
Potts–Guy equation, published coefficients	-0.172	-0.268	-0.215	-0.287	-0.098	-0.087
Potts–Guy functional form, refitted	0.223	0.078	0.213	0.080	0.180	0.170
Multiple linear regression, all descriptors	0.218	0.066	0.205	0.082	0.140	0.146
Ridge regression, all descriptors	0.201	0.144	0.203	0.144	0.164	0.162
Random forest	0.364	0.312	0.364	0.318	0.203	0.336
Gradient boosting	0.309	0.274	0.315	0.282	0.128	0.291

CV = cross-validated. Under the median and mean treatments each compound contributes one value and the unit of analysis is the compound (261 fitted, 66 held out); under the record-level treatment every individual measurement is retained (577 fitted, 155 held out) and cross-validation folds are grouped by compound, so no compound is divided between folds. The split is the same stratified 80:20 split on compounds throughout.

Table S2. Variance components of a mixed-effects model of log Kp at record level, with compound as a random intercept.

Fixed effects	Between-compound variance	Within-compound variance	ICC	SD between	SD within
Intercept only	0.862	0.928	0.482	0.928	0.963
Two descriptors (log P, molecular weight)	0.462	0.945	0.328	0.680	0.972
All fifteen descriptors	0.448	0.931	0.325	0.669	0.965

ICC = intraclass correlation coefficient, the share of record-level variance lying between compounds. Fitted on all 732 records by restricted maximum likelihood. The complement of the ICC in the intercept-only model, 0.518, is the share of variance lying between repeated measurements of the same compound.

Table S3. Distribution of held-out performance across 200 repeated stratified 80:20 splits.

Model	Median held-out R2	2.5th-97.5th percentile	SD	Median RMSE	First place (%)	Mean rank
Random forest	0.383	0.144 to 0.559	0.101	0.955	79.0	1.33
Gradient boosting	0.323	0.055 to 0.531	0.122	1.014	9.0	2.61
Potts–Guy functional form, refitted	0.240	0.078 to 0.377	0.077	1.058	4.5	3.56
Ridge regression, all descriptors	0.225	0.062 to 0.380	0.080	1.071	1.0	3.66
Multiple linear regression, all descriptors	0.220	-0.011 to 0.394	0.112	1.081	6.5	3.94
Potts–Guy equation, published coefficients	-0.119	-0.638 to 0.199	0.229	1.297	0.0	5.91

Models are ordered by mean rank. The percentile columns describe the variability of the estimate across splits and are not confidence intervals for a single split. First place is the percentage of splits in which the model had the highest held-out R2.

Table S4. Comparisons between descriptor sets and between skin-layer subsets, corrected for the dependence between cross-validation folds.

Comparison	Mean difference in R2	95% CI	Corrected t	Corrected p	Uncorrected p
Protocol descriptors vs molecular descriptors	0.096	-0.076 to 0.285	0.670	0.519	0.556641

Both sets vs molecular descriptors alone	0.178	0.101 to 0.249	3.065	0.013	0.005859
Both sets vs protocol descriptors alone	0.082	-0.102 to 0.252	0.581	0.576	0.492188
Epidermis only vs mixed skin layers	0.231	—	1.931	0.059	3.6e-06

Corrected t and p use the Nadeau-Bengio correction for resampled tests, which accounts for the training data shared between folds; the uncorrected column gives the test reported before correction (Wilcoxon signed-rank for the first three rows, Mann-Whitney U for the fourth). The skin-layer row compares 50 resampled evaluations and has no paired difference interval; its group means are 0.443 (95% CI 0.391 to 0.493) for epidermis and 0.213 (0.135 to 0.286) for mixed layers, Cohen d = 0.989.

Table S5. Effect of adding computed ionisation descriptors at pH 7.4 to the descriptor set.

Descriptor set	CV R2	SD	CV RMSE	Number of descriptors
Random forest, fifteen descriptors	0.364	0.183	0.960	15
Random forest, fifteen descriptors plus ionisation	0.367	0.194	0.956	18
Two descriptors (log P, molecular weight)	0.223	0.066	1.077	2
Two descriptors plus ionisation	0.228	0.098	1.074	5

Ionisation was computed with the site pKa library of Dimorphite-DL and the Henderson-Hasselbalch relation; the three added descriptors are the total fraction ionised and its anionic and cationic components. Ten-fold cross-validation on all 327 compounds.

Table S6. Composition of the ionisable set at pH 7.4, computed with the site pKa library of Dimorphite-DL.

Class at pH 7.4	Compounds	Median fraction ionised	Median fraction anionic	Median fraction cationic
No ionisable site	220	0.000	0.000	0.000
Basic	69	0.852	0.000	0.852
Acidic	23	0.999	0.999	0.000
Ampholytic	15	0.999	0.999	0.852
All compounds	327	0.000	0.000	0.000

Fraction ionised is the sum of the anionic and cationic fractions from the Henderson-Hasselbalch relation at pH 7.4. Compounds with no ionisable site in the library are counted as neutral. The three descriptors added to the set in Table S5 are the total fraction ionised and its anionic and cationic components.

Conformity with the OECD principles for the validation of (Q)SAR models

Prepared in response to Reviewer 2, Major 10. Every statement below refers to what was actually done in this study; where a principle is only partly met, that is stated rather than glossed.

---

1. A defined endpoint

The endpoint is the steady-state permeability coefficient of human skin, reported as log Kp in cm s^{-1}.

Values were taken from two open databases of *in vitro* human skin permeation, HuskinDB and SkinPiX version 1.1, and harmonised to a common unit; where a source reported cm h^{-1}, log$_{10}$(3600) was subtracted. Records measured on non-human skin were excluded by the source databases' own scope. The pooled analysis set is 732 records for 327 compounds.

Qualification. The endpoint is defined at the level of the measured quantity, not at the level of a single standardised protocol. The records were produced under heterogeneous conditions — skin layer, anatomical site, diffusion cell, donor and receptor temperature and pH — and this study treats that heterogeneity as an object of analysis rather than removing it by exclusion. A mixed-effects decomposition of the record-level variance places 52 per cent of it within compounds, that is between repeated measurements of the same molecule. Any model of this endpoint inherits that variability.

---

2. An unambiguous algorithm

Six predictors were compared, all fully specified:

Model	Specification
Potts–Guy (literature)	log Kp = −2.72 + 0.71 log K~ow~ − 0.0061 MW, published coefficients applied without fitting, converted to cm s^{-1}
Potts–Guy form, refitted	same two descriptors, coefficients refitted to the present data by ordinary least squares on standardised inputs
Multiple linear regression	ordinary least squares on all 15 standardised descriptors
Ridge regression	as above with an L2 penalty, the penalty selected by internal cross-validation within the training set only
Random forest	800 trees, minimum leaf size 2, all other scikit-learn defaults
Gradient boosting	700 trees, learning rate 0.03, maximum depth 4, subsample 0.8, column subsample 0.8, L2 regularisation 1.0

No hyperparameter search was performed. The ensemble settings were fixed a priori and never varied; the ridge penalty is the only quantity chosen from data, and it was chosen inside the training set. The held-out test set took no part in fitting, in penalty selection, or in any choice of setting.

Descriptors are 15 two-dimensional RDKit descriptors (version 2026.03.5): molecular weight, Crippen log *P*, molar refractivity, topological polar surface area, counts of hydrogen-bond donors and acceptors, rotatable bonds, aromatic rings, total rings, heavy atoms and heteroatoms, fraction of sp^3-hybridised carbon, Labute approximate surface area, Balaban J index and Bertz complexity index.

Random seed 20260821 throughout. Split assignments, grouped cross-validation fold assignments and the software versions are deposited alongside the code, so every split and fold can be reproduced exactly rather than re-estimated.

---

3. A defined domain of applicability

The applicability domain is the mean Euclidean distance, in standardised descriptor space, to the five nearest training compounds, with the threshold set at the 95th percentile of the corresponding distances within the training set.

All 40 analgesics in the leave-class-out analysis fall inside this domain.

Qualification, and it matters. Falling inside the domain did not make the predictions reliable at the edges. Predictions for the analgesic class were systematically compressed at both

extremes, over-predicting the least permeable opioids such as morphine and under-predicting the most permeable compounds, fentanyl and sufentanil — which are the clinically important members of the class. A domain check of this kind reports chemical similarity to the training set; it does not certify accuracy, and this study is an explicit example of the difference.

---

4. Appropriate measures of goodness-of-fit, robustness and predictivity

| Measure | How obtained | |---|---| | Cross-validated R^2 and RMSE | 10-fold cross-validation; folds grouped by compound wherever records rather than compounds are the unit, so no compound is split across folds | | Held-out R^2 , RMSE and MAE | one stratified 80:20 split on compounds, evaluated once | | Distribution of held-out R^2 | the split repeated 200 times under the same stratification; the random forest gives a median of 0.383 with a 2.5–97.5 percentile interval of 0.144 to 0.559, takes first place in 79 per cent of splits, and the single split reported in the manuscript sits at the 26th percentile of its own distribution | | Extrapolation to a new class | all 40 analgesics withheld from training, models refitted on the 287 remaining compounds | | Sensitivity to aggregation | repeated with the mean in place of the median, at record level with grouped cross-validation, and as a mixed-effects model with compound as a random intercept | | Fold-wise comparisons | Nadeau-Bengio correction for resampled tests, reported with effect sizes and intervals, because cross-validation folds share training data and are not independent | | Confounding check | permutation test preserving compound identity, and nearest-neighbour matching on standardised descriptors, for the skin-layer comparison |

Qualification. Two results weakened under these checks and are reported as weakened: the skin-layer difference is no longer significant after correction for fold dependence ($p = 0.059$, from $p = 0.000004$), and the molecular-weight threshold does not survive an accumulated-local-effects analysis, its span of 0.44 log units being smaller than the mean width of its own 95 per cent bootstrap band.

---

5. A mechanistic interpretation, if possible

A mechanistic reading is offered for one relationship and explicitly withheld for the other.

Offered. The dependence on lipophilicity is saturating: predicted log Kp rises steeply and plateaus above a computed octanol–water partition coefficient of about 2.5. Under accumulated local effects, which are robust to the collinearity among the descriptors, this spans 1.93 log units against a within-compound noise standard deviation of 0.963, so it is resolved by the data. It is consistent with partition-limited transport across the lipid-rich stratum corneum: once a solute partitions favourably into the intercellular lamellae, further lipophilicity adds little to the rate at which it crosses the barrier.

Withheld. The model also learned a decline in permeability above a molecular weight near 220 g mol^{-1}, and an earlier version of this manuscript presented it as a finding. It does not survive: the effect spans 0.44 log units against a bootstrap band of 0.54, molecular weight carries no information independent of the Labute approximate surface area (partial $r = 0.008$, $p = 0.88$), and removing molecular weight from the descriptor set moves cross-validated R^2 from 0.364 to 0.366. It is now reported as a feature of the fitted model rather than a property of skin.

Summary

Principles 1, 2 and 4 are met in full. Principle 3 is met as specified, with the limitation that domain membership did not guarantee accuracy at the extremes of the held-out class. Principle 5 is met for the lipophilicity relationship and deliberately not claimed for molecular weight.
